# Chlorine Water in the Treatment of Diphtheria

**Published:** 1873-01

**Authors:** W. G. Balfour

**Affiliations:** Assistant Medical Officer to the Montrose Royal Asylum


### SELECTIONS.

### CHLORINE WATER IN THE TREATMENT OF DIPHTHERIA.

BY W. G. BALFOUR, L.R.C.S.E.,

*Assistant Medical Officer to the Montrose Royal Asylum.* (*Edinburgh Med. Jour., Dec.*, 1871.)

Chlorine water is used by many practitioners in the treatment of scarlet fever. In the Medical Times and Gazette, of the 25th of August, 1869, there is a letter by Messrs. Taynton and Wells, stating that they employed chlorine water in cases of scarlet fever, and they "most solemnly declare that it proved successful in almost every case to which they were called in time, and in which the medicine was *faithfully* administered." At page 209 of Watson's Practice of Physic, vol. ii. (fourth edition), mention is made of this letter in the Medical Times and Gazette; and the good effect of chlorine water is there stated to depend upon its disinfecting properties, probably depriving the secretions from the throat in scarlet fever of their noxious qualities.*

Dr. Matthew Gairdner, of Crieff, was, as far as I know, the first to use chlorine water in diphtheria, with the best results; and it was while acting as his assistant that I became aware of its good effects.

The following observations are made with the hope that an extended trial of the chlorine solution in dipththeria may establish its value in that disease:

In a family in the neighborhood of Crieff, four of the children suffered from well-marked diphtheritic sore-throat. In all of them the tonsils and pharynx presented patches of diphtheritic membrane, the sub-maxillary glands were enlarged, and the urine in two of the cases contained albumen. They were all treated with chlorine water, stimulants and milk. Three of the children recovered; the fourth died. Dr. Ormond saw the child that died along with me, and confirmed my opinion as to the nature of the disease. I attributed this death to the fact that the mother failed to *faithfully* administer the medicine, assigning afterwards, as her reason, that it made "the poor thing cry."

The conclusion that the want of the medicine was the cause of the child's death may have been wrong; but seeing those who took

*To prepare chlorine water, put grs. viii. of potas. chlor. in a strong pint bottle; add dr. i. of acid hydrochlor. fort.; close the mouth of the bottle whilst the violent action lasts; then add water, ounce by ounce, with constant agitation, till the bottle is full. (An adult may use the whole pint in a day.)

it all recovered, and that their symptoms at the beginning were fully as severe as those of the child that died, it is probable.

The following severe case of diphtheria, met with recently, appears strongly to confirm the good effects I formerly obtained in the treatment of this disease by chlorine in solution:

B. W., a child three years of age, complained one morning of a sore-throat, and on examination there was seen a deposit of false membrane, the size of a three-penny piece, on the right tonsil. Diphtheria prevailed in the neighborhood, and although there was no proof of contagion, I had strong suspicions that it was a case of diphtheria. A solution of potas. chlor. in water was given. Next day there was a further deposit of false membrane and marked constitutional symptoms.

The breathing was short, gasping and difficult; the pulse 130 per minute, and the urine albuminous; a teaspoonful vin. ipecac. afforded temporary relief to the breathing; but in three hours after the administration of the emetic, the symptoms again becoming urgent, recourse was had to the chlorine solution in ʒ ii. doses every two hours.

Next day the symptoms were, if anything, more intense than before, the urine highly albuminous; all solid food was refused; milk and wine in small quantities and the chlorine solution were, however, still taken.

By the evening the child was still breathing in gasps, and evidently fast sinking. Hot fomentations were applied to the throat and chest, more with the object of doing something than from any hope of good resulting. Shortly after the application to the throat, the child had a paroxysm of coughing, followed by a profuse expectoration of something, which was swallowed (probably the false membrane, as there was immediate relief to the breathing.)

The child steadily improved after this, the chlorine being continued for several days.

A week after convalescence was established, the child choked whilst drinking some water, part of the fluid being ejected through the nostrils. This choking occurred at times for several weeks after convalescence was established, and there was also aphonia.

Other cases of diphtheria, treated in exactly the same way whilst under Dr. Gairdner, of Crieff, might be published. The one I have given is, in itself, so typical, and its termination so satisfactory, that any addition would be superfluous for the purpose in view, viz: that of calling the attention of the profession to the value of chlorine in diphtheria.

The remedial action of chlorine in diphtheria and scarlet fever appears to be more general than local. It cannot exert much influence on the exudations, which are too often in the larynx and trachea, and consequently beyond its reach when the medicine is taken in solution.

Chlorine in solution, when taken internally, is absorbed as chlorine into the blood; in poisonous doses, producing decomposition of the blood corpuscles and hyperchlorite of soda. If, then, dipththeria be a disease due to toxæmia, it seems highly probable that chlorine, being absorbed into the blood even in small quantities, can hardly fail to affect the poison present in the system in some way.

The case given seems strongly to support this view, for it will be observed that, before the chlorine was administered, the constitutional and local symptoms were so severe as to lead to a prognosis the reverse of favorable.

There was evidently a large deposit of false membrane in the trachea and larynx, and until this was got rid of by a fit of coughing, death from suffocation was imminent.

There was no fresh deposit of membrane after the coughing, but whether due to the chlorine or not, others must judge.

Whatever be the theory of the action of chlorine in cases of dipththeria, I have nót found it fail in the treatment of this disease, when faithfully administered, since Dr. Gairdner called my attention to it, and I trust others may be induced to try it with equally favorable results.—*American Journal of Obstetrics.*

